# The role of the built environment in explaining educational inequalities in walking and cycling among adults in the Netherlands

**DOI:** 10.1186/s12942-017-0083-y

**Published:** 2017-03-31

**Authors:** Daniël C. van Wijk, Joost Oude Groeniger, Frank J. van Lenthe, Carlijn B. M. Kamphuis

**Affiliations:** 1 Department of Human Geography and Spatial Planning, Faculty of Geosciences, Utrecht University, Heidelberglaan 2, 3584 CS Utrecht, The Netherlands; 2 Department of Public Health, Erasmus University Medical Centre, Erasmus University Rotterdam, Wytemaweg 80, 3015 CN Rotterdam, The Netherlands

**Keywords:** Walking, Cycling, Built environment, Neighborhood, Health inequalities, GIS

## Abstract

**Background:**

This study examined whether characteristics of the residential built environment (i.e. population density, level of mixed land use, connectivity, accessibility of facilities, accessibility of green) contributed to educational inequalities in walking and cycling among adults.

**Methods:**

Data from participants (32–82 years) of the 2011 survey of the Dutch population-based GLOBE study were used (N = 2375). Highest attained educational level (independent variable) and walking for transport, cycling for transport, walking in leisure time and cycling in leisure time (dependent variables) were self-reported in the survey. GIS-systems were used to obtain spatial data on residential built environment characteristics. A four-step mediation-based analysis with log-linear regression models was used to examine to contribution of the residential built environment to educational inequalities in walking and cycling.

**Results:**

As compared to the lowest educational group, the highest educational group was more likely to cycle for transport (RR 1.13, 95% CI 1.04–1.23), walk in leisure time (RR 1.12, 95% CI 1.04–1.21), and cycle in leisure time (RR 1.12, 95% CI 1.03–1.22). Objective built environment characteristics were related to these outcomes, but contributed minimally to educational inequalities in walking and cycling. On the other hand, compared to the lowest educational group, the highest educational group was less likely to walk for transport (RR 0.91, 95% CI 0.82–1.01), which could partly be attributed to differences in the built environment.

**Conclusion:**

This study found that objective built environment characteristics contributed minimally to educational inequalities in walking and cycling in the Netherlands.

**Electronic supplementary material:**

The online version of this article (doi:10.1186/s12942-017-0083-y) contains supplementary material, which is available to authorized users.

## Background

The twenty-first century is going to be an urban one. It is estimated that 54% of the world’s population was living in urban areas by the year of 2014, and this figure will rise steadily over the following decades [1]. While urban areas are now believed to be of great importance in sustaining economic growth, it is less clear-cut what the consequences of this urban growth will be for population health. Living in an urban area may have positive as well as negative health consequences (e.g., urban areas offer better accessibility to various health-promoting resources, but in most cases have worse air quality), resulting in the use of the concepts of an ‘urban health advantage’ and an ‘urban health penalty’ simultaneously [2].

One of the key aspects of a healthy lifestyle is enough physical activity; 30 min of moderate-intensity activity a day is widely believed to have great health benefits [3, 4]. One way to promote physical activity is by facilitating the use of active travel modes (i.e. walking and cycling), either for transport-related or for recreational purposes. Increasing walking and cycling levels seems an attractive option, because walking and cycling are accessible options to (almost) everyone and can be easily integrated into an individuals’ daily activity program.

It is becoming increasingly clear that spatial planning has an important role to play in the promotion of a healthy urban lifestyle. While personal factors are critical in determining individual health, the built environment has the potential to exacerbate or mitigate health outcomes for large populations groups [5]. Various studies suggest a relationship exists between the built environment and the amounts of walking and cycling [6]. The built environment can thus play a vital role in promoting a healthy, physically active, urban lifestyle.

An important theme in health policy is the reduction of health inequalities [7]. Unhealthy lifestyles tend to be present more in lower socioeconomic groups, resulting in poorer health and higher mortality rates [8]. This general relationship is, however, less obvious in the case of physical activity, where the direction of socioeconomic inequalities seems to differ considerably by domain. A high socioeconomic position is related to higher levels of leisure-time physical activity [9], but socioeconomic inequalities in active transport do not show a consistent pattern [10]. Although the built environment is often presumed to be an explanatory factor of socioeconomic inequalities in walking and cycling, little research has yet investigated this issue. Because place of residence is strongly patterned by socioeconomic position, the neighborhood could be an important contributor to socioeconomic inequalities in health [11, 12]. Yet, pathways may be less straightforward: exposure to more facilities for example may increase walking for transport purposes, but decrease walking in leisure time. Moreover, if and to what extent higher and lower socioeconomic groups are differentially exposed to built environmental characteristics requires further investigation.

The Netherlands offer an interesting study setting for this issue, because Dutch cities are generally characterized by a relatively dense urban context, which is more conducive to walking and cycling [13].

**Fig. 1:**
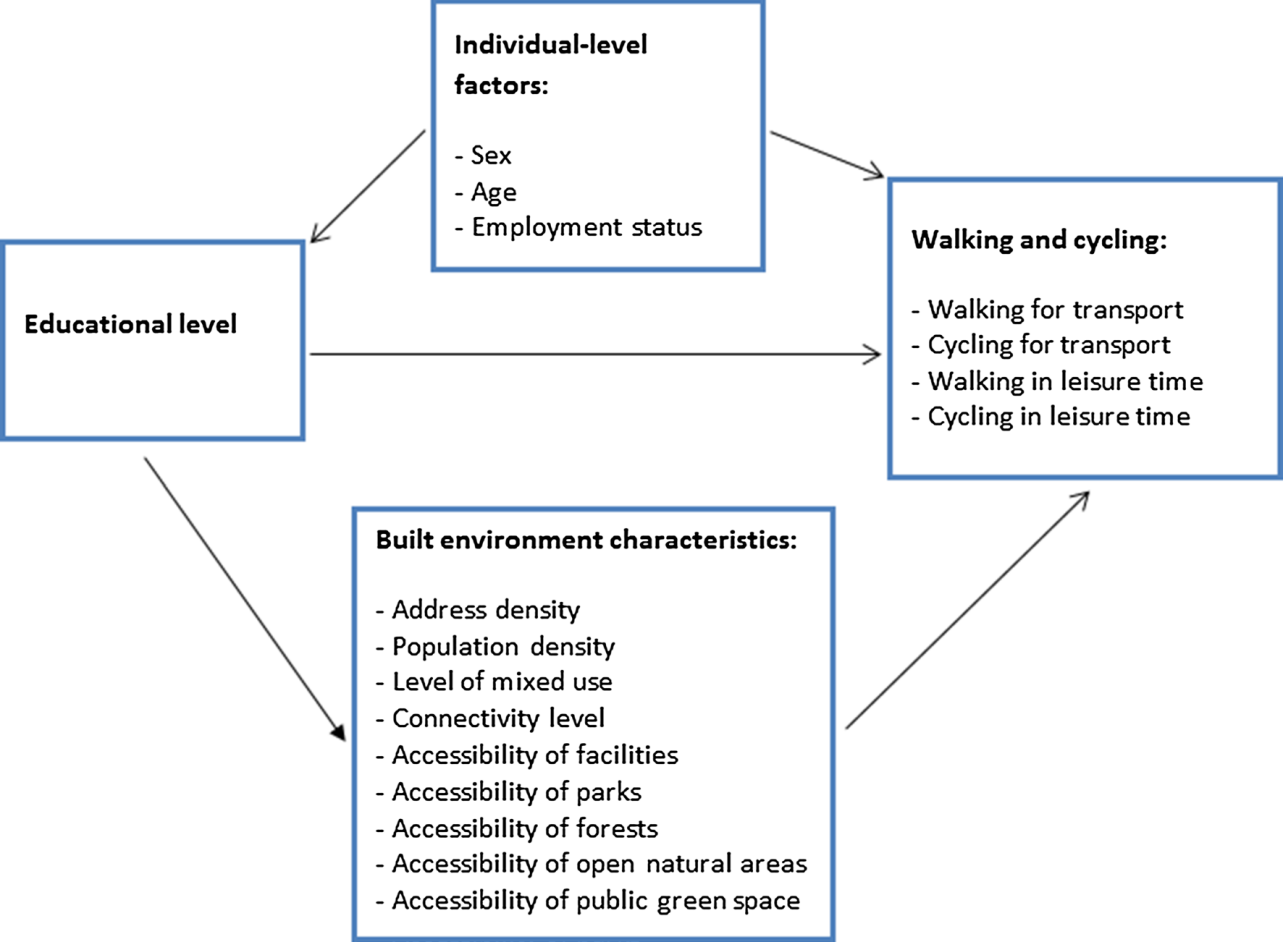
Conceptual model of the association between educational level, built environment and walking and cycling

Therefore the overall aim of this study is to examine to what extent objective built environment characteristics contribute to socioeconomic inequalities in walking and cycling—both transport-related and in leisure time—in the Netherlands. More specifically, we investigate (1) to what extent educational inequalities in walking and cycling exist in the Netherlands, (2) to what extent higher and lower educational groups reside in neighborhoods with different objective built environment characteristics, i.e. density, level of mixed use, connectivity, accessibility of facilities and accessibility of green, (3) the associations of these built environment characteristics with walking and cycling, and (4) to what extent these built environment characteristics contribute to the explanation of educational inequalities in walking and cycling in the Netherlands. Figure 1 schematically represents the relationships between the different factors that are examined in this study.

## Methods

### Study population

This study uses survey data from the GLOBE cohort study, collected among adults living in the Dutch region of Eindhoven and surroundings in the year 2011. The GLOBE study is a cohort study that started in 1991. The city of Eindhoven and its surrounding villages was chosen as study location, because its composition was reasonably representative for the Netherlands in terms of age, sex, and educational level. Follow-up data collections were conducted in 1997, 2004, 2011 and 2014 (for more information on the GLOBE study, see [14]). This study uses data from the GLOBE survey of 2011 (N = 2888; mean age 60.69 (SD = 13.27); range 32–91 years). The 2011 survey was chosen because this survey contains the most detailed information on various sorts of walking and cycling activities. Respondents who had missing data for educational level, sex, age, employment status, or built environment variables (N = 513; some neighborhoods had missing data on accessibility of green variables due to neighborhood reorganizations since 2008) were excluded from the analysis, reducing the sample to N = 2375, residing in N = 209 neighborhoods (the neighborhood is defined in section “Built environment characteristics of the neighborhood”).

### Walking and cycling

Walking and cycling activity was measured using the SQUASH questionnaire [15]. Respondents were asked how many days per week they walked and cycled (1) in leisure time, (2) to shops and other facilities, and (3) to work or school. Walking to shops and other facilities and walking to work or school were grouped together to compose a ‘walking for transport’ variable. The same was done for cycling to shops and other facilities and cycling to work or school. For each of the outcome variables, a high share of the respondents indicated to walk ‘0 days per week’. Therefore, each outcome was dichotomized in either walking/cycling at least once per week versus no weekly walking/cycling. Thus, four dichotomous variables were created: walking for transport (yes/no) cycling for transport (yes/no), walking in leisure time (yes/no), and cycling in leisure time (yes/no).

### Educational level

Educational level was used as indicator of socioeconomic position. A study by Winkleby et al. [16, see also 17] showed that education was the best socioeconomic predictor of good health. Also, in the 2011 GLOBE survey, educational level was characterized by high response rates (i.e. higher than income). In the 2011 survey, information of respondents’ educational level was acquired through questions on highest education completed (with options ranging from no education to university education). This information was grouped into three categories, based on a categorization used by Statistics Netherlands [18]. The ‘low’ educational group comprised respondents with no education and respondents who completed only primary education or lower secondary education (ISCED 0–2). The ‘middle’ educational group comprised respondents who completed either ‘middle-level applied education’ or higher secondary education (ISCED 3–4). The ‘high’ educational group comprised respondents who completed higher vocational education or university education (ISCED 5–7).

### Built environment characteristics of the neighborhood

The spatial level on which the built environment data for this study were collected was the neighborhood level, which is the smallest geographical unit used in the Netherlands for statistical purposes. In 2013, neighborhoods included in this study had an average population of 2558 (range 25–10,355) and an average size of 179 hectares (range 13–4243) [19]. The neighborhood level was deemed appropriate because it was expected to be of sufficient size to be of significant importance for people’s daily walking and cycling activities, yet small enough for local variation in exposures and outcomes to exist. Also, Statistics Netherlands frequently uses the neighborhood level to collect spatial data, which were used in this study.

In general, built environment aspects that seem to be of significant importance for walking and cycling are population density, street connectivity, accessibility of facilities, a mixed land use, greenery, aesthetics, the presence of recreational facilities such as parks and the availability of walking and cycling paths [6, 20]. However, due to data availability not all aspects could be included in this study. Also, characteristics can be operationalized in different ways, sometimes yielding different results (see, for example, [21]). Table 1 gives an overview of the built environment variables that were used in this study, and their sources. The variables used in the study partly measured the same characteristics, and some are expected to be highly correlated (e.g., a neighborhood with high levels of mixed use is expected to be more accessible). The combination of various factors might, however, provide additional explanatory power [22]. Below the calculations of all variables are described. Because of their distributional characteristics (i.e. highly skewed with some extreme outliers), for the analyses, the values of all built environment variables have been grouped into tertiles (low, medium, high).

**Table 1:** Description and source of all built environment variables used in the study

Variable	Description	Mean (SD)	Range	Data source
Address density	Degree of concentration by number of addresses within a 1 km radius on January 1, 2013	1533 (779.6)	16–3684	Statistics Netherlands [19]
Population density	Number of residents per km^2^ in a neighborhood on January 1, 2013	3999.3 (1904.7)	8–11,151	Statistics Netherlands [19]
Level of mixed use	Degree of mixed use of a neighborhood, measured by an entropy measure containing the categories ‘residential’ and ‘other’ in 2013	0.517 (0.203)	0.069–1	Kadaster [41]
Connectivity	Number of intersections per km^2^ in a neighborhood in November 2012	140.2 (51.5)	2–303	Kadaster [42]
Accessibility of facilities	Number of facilities within a 1 km radius in 2013. The following facilities were used to calculate this variable (weights between brackets): big supermarkets (10), other daily provisions (5), cafes (1), cafeteria (1), restaurants (1), nurseries (1), out-of-school care (1) and schools (5)	68.9 (61.8)	0–424	Statistics Netherlands [19]
Accessibility of parks	Accessibility measure based on the mean distance to a park for all residents of a neighborhood in 2008	0.613 (0.544)	0.1–4.6	Statistics Netherlands [30]
Accessibility of forests	Accessibility measure based on the mean distance to a forest for all residents of a neighborhood in 2008	1.078 (0.512)	0.2–2.7	Statistics Netherlands [30]
Accessibility of open natural areas	Accessibility measure based on the mean distance to an open natural area for all residents of a neighborhood in 2008	3.197 (1.442)	0.5–6.3	Statistics Netherlands [30]
Accessibility of public green space	Accessibility measure based on the mean distance to public green space (i.e. one of the above) for all residents of a neighborhood in 2008	0.441 (0.196)	0.1–1.2	Statistics Netherlands [30]

*Address density* was derived from Statistics Netherlands [19] and comprised the mean number of addresses per km^2^ within a 1 km radius of each address within the neighborhood. For the calculation of this variable, first, the address density for each address (x–y- coordinate) in the neighborhood was calculated. Then the mean address density of the neighborhood was calculated by dividing the sum of all address densities in the neighborhood by the total number of addresses in the neighborhood [23].

*Population density* was calculated by Statistics Netherlands [19] as the number of residents of a neighborhood divided by the total land area of the neighborhood. A first difference between population density and address density is that address density contains all sorts of human activity (e.g. shops, offices), whereas population density only contains residential activity. A second difference is the geographic area of measurement: while the population density variable comprised the density of the neighborhood area, the address density variable includes all addresses in a 1 km radius. This difference in calculation may yield substantial differences in outcomes. For example, a densely populated neighborhood located at the edge of a city will be characterized by high levels of population density, but low levels of address density.

The *level of mixed use* was based on the distribution of land use in an area. It was calculated using an entropy equation to form a measure that ranges from 0 to 1, with 0 representing complete homogeneity of land use and 1 representing an even distribution of all types of land use [24, 25]. In the present study, the level of mixed use was not calculated using proportions of land area. Instead, the type of land use was derived from individual addresses. The variable can therefore be seen as the degree of mixing of different address types. Because of the high proportion of addresses in the study area that were characterized as residential, the entropy measure is based solely on two categories: ‘residential’ and ‘other’ (e.g. retail, industry). It was calculated using the following equation (adapted from [26]):

$$- 1\frac{{\frac{proportion\;'residential'}{total\;number\;of\;addresses} \times \ln \left( {\frac{proportion\;'residential'}{total\;number\;of\;addresses}} \right) + \frac{proportion\;'other'}{total\;number\;of\;addresses} \times \ln \left( {\frac{proportion\;'other'}{total\;number\;of\;addresses}} \right)}}{\ln \left( 2 \right)}$$

The level of *connectivity* was calculated as the sum of intersections with at least three converging roads or pathways in a neighborhood, divided by the size of the neighborhood to control for differences in neighborhood size. For the purpose of this study, intersections that were primarily used by cars (e.g. highways, provincial roads) were excluded. This measure has been used previously [22, 25, 27], where it was shown to be a significant predictor of walking and cycling.

The *accessibility of facilities* was based on data derived from Statistics Netherlands [19] containing the mean number of various facility types within a road distance of 1 km. This was calculated using the number of facilities within a road distance of 1 km from every address in the neighborhood, divided by the total number of addresses [23]. Weights were assigned by the authors based on their expected frequency of visits (see Table 1). This variable can be seen as an elaboration of the ‘cumulative opportunities’ measure distinguished by Handy and Niemeier [28], which emphasizes the number of potential opportunities within a given travel distance (see also [29]).

The four accessibility of green variables (i.e. *accessibility of parks*, *accessibility of forests*, *accessibility of open natural areas*, and *accessibility of public green space*) were derived from Statistics Netherlands [30] and were calculated using data on the mean distance of all residents of a neighborhood to the closest park. For reasons of interpretation, after categorization this figure was ‘swapped’ (i.e. the lowest scores were turned into the highest and vice versa) to turn ‘distance’ into ‘accessibility’. The *accessibility of public green space* variable is a combination of the other three accessibility of green variables, based on the mean distance of all residents of a neighborhood to the closest green space.

### Statistical analysis

We applied a four-step mediation-based analysis approach, largely similar to previous studies [25]. In all steps, the models were adjusted for potential confounders, i.e. age, sex and employment status (the categorization used, and the distribution of respondents across these control variables, are shown in Table 2). In the first step, walking and cycling for different purposes (the dependent variables) were separately regressed on educational level (the independent variable). Second, educational level was separately correlated with each built environment variable. Third, each walking and cycling variable was separately regressed on each of the built environment variables, adjusted for educational level. Fourth, all built environment variables that were significant in the third analysis were added to the regression of the different walking and cycling variables on educational level. Comparison between the first and fourth analysis showed the contribution of the built environment variables to educational differences in walking and cycling levels [25].

**Table 2:** Distribution of respondents across variables in different educational groups (N = 2375)

Sex	Low education		Middle education		High education		Total	
	N	%	N	%	N	%	N	%
Female	674	65.4	277	54.7	363	48.2	1314	56.3
Male	354	34.6	283	45.3	424	51.8	1061	43.7
Age								
30–39	22	3.5	70	15.0	114	18.1	206	11.9
40–49	68	9.9	138	29.1	165	24.0	371	20.2
50–59	140	21.7	113	24.0	172	24.3	425	23.3
60–69	331	36.2	137	21.3	175	21.1	643	26.7
70–79	405	24.6	84	8.6	144	10.8	633	15.2
80–89	62	4.0	18	2.1	17	1.7	97	2.7
Employment status								
Full-time	97	15.4	151	32.0	258	38.0	506	28.1
Part-time	93	13.8	147	30.8	192	29.3	432	24.0
Not working	838	70.7	262	37.2	337	32.7	1437	47.8
Walking for transport								
No	368	38.2	197	37.2	321	41.8	886	39.2
Yes	660	61.8	363	62.8	466	58.2	1489	60.8
Cycling for transport								
No	445	36.7	188	33.2	219	27.1	852	32.3
Yes	583	63.3	372	66.8	568	72.9	1523	67.7
Walking in leisure time								
No	352	31.6	124	21.7	173	22.6	649	25.6
Yes	676	68.4	436	78.3	614	77.4	1726	74.4
Cycling in leisure time								
No	419	35.9	167	29.6	206	26.2	792	30.7
Yes	609	64.1	393	70.4	581	73.8	1583	69.3

Frequencies are not weighted, percentages are

The second step in the analysis (correlation between educational level and built environment variables) was conducted using crosstabs and Kendall’s Tau-b (conducted in SPSS 22). All other analyses were conducted using multilevel log-linear analysis (conducted in Stata 14). Although in all analyses the variance at the neighborhood level was very small, multilevel models were nevertheless used to account for the clustering of individuals within neighborhoods. Log-linear regression instead of logistic regression was used because the distribution of respondents across the dependent variables was characterized by relatively high numbers of respondents in the ‘no walking/cycling’ groups (i.e. more than 10%), raising the problem of ‘non-collapsibility’ of odds ratios in a logistic regression mediation analysis. Log-linear analysis—which uses risk ratios instead of odds ratios—was used to tackle this problem [31].

All statistical analyses were weighted to be representative for the population of Eindhoven and surroundings aged 25–75 in the year 2004.

## Results

A description of demographic characteristics and an initial examination of the distribution of walking and cycling variables among the three educational groups can be found in Table 2. This table shows that respondents in the low educational category were more frequently female (65.4%), older than 60 (64.8%), and not working (70.7%) than respondents in the higher educational categories (48.2, 33.8, and 32.7%, respectively, for the high educational category). Also, walking and cycling were in all cases practiced by more than half of the respondents.

 In addition, maps of the various built environment variables and of the spatial distribution of respondents by educational level can be found in the Additional file 1: Appendix. These maps show that most built environment variables (both density variables, connectivity, accessibility of facilities and accessibility of parks) had higher levels in the Eindhoven city center and other urban centers in the area (those of Best and Helmond), while a few built environment variables (level of mixed use, accessibility of forests and accessibility of open natural areas) showed higher values in more suburban and rural areas. A correlation analysis between built environment variables (see Additional file 1: Appendix) showed that none of the paired variables showed problematically high correlation outcomes [the highest correlations were found between accessibility of parks and accessibility of public green (*τ*_B_ = 0.65) and between population density and connectivity (*τ*_B_ = 0.63)], so each variable can be assumed to contain unique characteristics.

### Educational level and walking and cycling

The highest educational group was more likely to cycle for transport (RR 1.13, 95% CI 1.04–1.23) walk in leisure time (RR 1.12, 95% CI 1.04–1.21) and cycle in leisure time (RR 1.12, 95% CI 1.03–1.22) than its low-educated counterpart. Respondents in the group with the highest education were less likely to walk for transport (RR 0.91, 95% CI 0.82–1.01) than their lower-educated counterparts, but this association was not significant (see Table 3).

**Table 3:** Associations between educational level and walking and cycling for transport and in leisure time (N = 2375)

Educational level	RR (95% CI)	Sig.
Walking for transport		
Low	1.00	0.152
Middle	0.98 (0.86–1.10)	
High	0.91 (0.82–1.01)	
Cycling for transport		
Low	1.00	0.009
Middle	1.02 (0.93–1.12)	
High	1.13 (1.04–1.23)	
Walking in leisure time		
Low	1.00	0.006
Middle	1.12 (1.04–1.21)	
High	1.12 (1.04–1.21)	
Cycling in leisure time		
Low	1.00	0.021
Middle	1.07 (0.98–1.16)	
High	1.12 (1.03–1.22)	

All analyses were adjusted for variations in sex, age and employment status

### Educational level and built environment

There was a weak but significant inverse association between educational level and address density (*τ*_B_ = −0.065), educational level and population density (*τ*_B_ = −0.037), educational level and level of mixed use (*τ*_B_ = −0.080), educational level and accessibility of facilities (*τ*_B_ = −0.059), and a positive association between educational level and accessibility of forests (*τ*_B_ = 0.036). No significant association was found between educational level and connectivity, accessibility of parks, accessibility of open natural areas, and accessibility of public green space.

### Built environment and walking and cycling

Residents of neighborhoods with higher levels of address density, population density, connectivity and accessibility of facilities were more likely to walk for transport than residents of neighborhoods with the lowest scores, all significant at the 5% level. For example, residents of neighborhoods with the highest level of address density were 1.33 times more likely to walk for transport than residents of neighborhoods with the lowest scores (RR 1.33, 95% CI 1.21–1.47; see Table 4 for more results). Walking for transport was less prevalent among residents of neighborhoods that were close to a forest [RR 0.87, 95% CI 0.78–0.96 (high category)]. No significant impact on walking for transport was found for the other accessibility of green variables and for the level of mixed use variable. Residents of neighborhoods with higher levels of population density were less likely to cycle for transport [RR 0.89, 95% CI 0.81–0.98 (high category)].

**Table 4:** Associations between walking and cycling and built environment (N = 2375)

	Walking for transport		Cycling for transport		Walking in leisure time		Cycling in leisure time	
	RR (95% CI)	Sig.	RR (95% CI)	Sig.	RR (95% CI)	Sig.	RR (95% CI)	Sig.
Address density								
1 (low)	1.00	0.000	1.00	0.293	1.00	0.005	1.00	0.004
2	1.04 (0.93–1.16)		0.96 (0.86–1.06)		0.90 (0.85–0.96)		0.90 (0.83–0.98)	
3 (high)	1.33 (1.21–1.47)		0.92 (0.84–1.02)		0.99 (0.94–1.04)		0.89 (0.82–0.96)	
Population density								
1 (low)	1.00	0.001	1.00	0.049	1.00	0.781	1.00	0.048
2	1.07 (0.97–1.19)		0.96 (0.86–1.06)		0.99 (0.93–1.05)		0.96 (0.89–1.04)	
3 (high)	1.22 (1.10–1.35)		0.89 (0.81–0.98)		1.01 (0.94–1.08)		0.91 (0.84–0.98)	
Level of mixed use								
1 (low)	1.00	0.159	1.00	0.549	1.00	0.821	1.00	0.740
2	1.10 (1.00–1.21)		1.04 (0.96–1.13)		1.02 (0.95–1.08)		1.03 (0.95–1.12)	
3 (high)	1.05 (0.94–1.18)		1.00 (0.89–1.12)		1.02 (0.95–1.09)		1.02 (0.94–1.11)	
Connectivity								
1 (low)	1.00	0.027	1.00	0.505	1.00	0.966	1.00	0.284
2	1.10 (0.997–1.22)		0.95 (0.86–1.06)		1.00 (0.94–1.07)		1.00 (0.92–1.08)	
3 (high)	1.14 (1.03–1.26)		0.94 (0.86–1.04)		1.01 (0.94–1.07)		0.94 (0.87–1.02)	
Accessibility of facilities								
1 (low)	1.00	0.000	1.00	0.890	1.00	0.037	1.00	0.959
2	1.09 (0.98–1.21)		1.02 (0.93–1.12)		0.96 (0.90–1.03)		1.01 (0.93–1.10)	
3 (high)	1.26 (1.13–1.41)		1.01 (0.91–1.12)		1.04 (0.99–1.11)		1.00 (0.92–1.08)	
Accessibility of parks								
1 (low)	1.00	0.354	1.00	0.530	1.00	0.632	1.00	0.538
2	1.03 (0.94–1.14)		1.00 (0.91–1.11)		1.01 (0.96–1.07)		0.99 (0.91–1.07)	
3 (high)	0.95 (0.86–1.06)		0.96 (0.86–1.07)		0.98 (0.91–1.05)		0.96 (0.88–1.04)	
Accessibility of forests								
1 (low)	1.00	0.015	1.00	0.218	1.00	0.758	1.00	0.592
2	0.89 (0.80–0.99)		1.08 (0.98–1.19)		0.98 (0.92–1.05)		1.03 (0.95–1.12)	
3 (high)	0.87 (0.78–0.96)		1.07 (0.98–1.17)		0.98 (0.91–1.04)		1.04 (0.96–1.13)	
Accessibility of open natural areas								
1 (low)	1.00	0.605	1.00	0.603	1.00	0.378	1.00	0.088
2	0.95 (0.85–1.06)		1.05 (0.96–1.15)		1.01 (0.95–1.08)		1.08 (1.01–1.17)	
3 (high)	0.96 (0.88–1.06)		1.02 (0.93–1.13)		0.96 (0.90–1.03)		1.02 (0.94–1.11)	
Accessibility of public green space								
1 (low)	1.00	0.379	1.00	0.539	1.00	0.292	1.00	0.040
2	0.95 (0.86–1.04)		0.95 (0.87–1.04)		0.99 (0.94–1.04)		0.93 (0.87–1.00)	
3 (high)	0.92 (0.79–1.06)		0.97 (0.84–1.11)		0.92 (0.83–1.02)		0.89 (0.80–0.99)	

All built environment variables were analyzed separately from each other

All analyses were adjusted for variations in sex, age, employment status and educational level

Residents of neighborhoods with higher levels of address density were less likely to walk in leisure time [RR 0.90, 95% CI 0.85–0.96 (middle); RR 0.99, 95% CI 0.94–1.04 (high)]. Next to this, a significant association was found between accessibility of facilities and walking in leisure time, which was negative for the middle group but positive for the high one [RR 0.96, 95% CI 0.90–1.03 (middle); RR 1.04, 95% CI 0.99–1.11 (high). Also, when compared to their counterparts of neighborhoods in the lowest density category, residents of neighborhoods with higher levels of address density (RR 0.90, 95% CI 0.83–0.98 (middle); RR 0.89, 95% CI 0.82–0.96 (high)] and population density (RR 0.96, 95% CI 0.89–1.04 (middle); RR 0.91, 95% CI 0.84–0.98 (high)] were less likely to cycle in leisure time. Lastly, residents of neighborhoods that were close to public green space were less likely to cycle in leisure time [RR 0.93, 95% CI 0.87–1.00 (middle); RR 0.89, 95% CI 0.80–0.99 (high); see Table 4].

### Educational level, objective built environment, and walking and cycling

The negative association between educational level and walking for transport attenuated after adjustment for built environment variables [RR 1.01, 95% CI 0.90–1.13, ΔRR +0.03 (middle educational group), RR 0.94, 95% CI 0.85–1.04, ΔRR +0.03 (high)]. Adjustment for built environment variables had only minimal effect on the associations between educational level and cycling for transport [RR 1.01, 95% CI 0.93–1.11, ΔRR −0.01 (middle), RR 1.12, 95% CI 1.03–1.22, ΔRR −0.01 (high)] and educational level and cycling in leisure time [RR 1.05, 95% CI 0.97–1.14, ΔRR −0.01 (middle), RR 1.11, 95% CI 1.02–1.20, ΔRR −0.01 (high)], and no effect was found for the association between educational level and walking in leisure time. After adjustment for built environment variables, middle and high educational groups were still significantly more likely to cycle for transport and to walk and cycle in leisure time than their counterparts in the low educational group (see Table 5).

**Table 5:** Associations between educational level and walking and cycling adjusted for significant built environment variables

Educational level	RR (95% CI)	Sig.	ΔRR
Walking for transport^a^			
Low	1.00	0.262	
Middle	1.01 (0.90–1.13)		+0.03
High	0.94 (0.85–1.04)		+0.03
Cycling for transport^b^			
Low	1.00	0.014	
Middle	1.01 (0.93–1.11)		−0.01
High	1.12 (1.03–1.22)		−0.01
Walking in leisure time^c^			
Low	1.00	0.004	
Middle	1.12 (1.04–1.21)		0.00
High	1.12 (1.04–1.21)		0.00
Cycling in leisure time^d^			
Low	1.00	0.039	
Middle	1.05 (0.97–1.14)		−0.01
High	1.11 (1.02–1.20)		−0.01

The changes in risk ratios (ΔRR) compare the risk ratios after adjustment for built environment variables to the risk ratios before adjustment for built environment variables (see Table 3)

^a^Adjusted for variations in sex, age, employment status, address density, population density, connectivity, accessibility of facilities and accessibility of forests

^b^Adjusted for variations in sex, age, employment status and population density

^c^Adjusted for variations in sex, age, employment status, address density and accessibility of facilities

^d^Adjusted for variations in sex, age, employment status, address density, population density and accessibility of public green space

## Discussion

Lower educational groups were less likely to cycle for transport and to walk and cycle in leisure time, but only minimal effects of mediating built environment variables were found. On the other hand, lower educational groups were more likely to walk for transport, which could partly be attributed to differences in the built environment. These results suggest that neighborhood density, level of mixed use, connectivity, accessibility of facilities and accessibility of green only make a small contribution to educational inequalities in walking and cycling behavior in the Netherlands.

### Explaining educational inequalities in walking and cycling

Walking for transport was the only outcome that respondents with higher educational levels were less likely to perform. This study showed that this relationship attenuated after adjustment for built environment variables, indicating that respondents with a low educational level were more likely to walk for transport partly because their residential built environment was more conducive to this activity type. This is consistent with previous findings by Turrell et al. [25], who found that higher levels of walking for transport among residents of poorer neighborhoods in Australia could partly be explained by the higher connectivity, density and land use mix levels in those neighborhoods. It is also consistent with findings of a review of American studies, which found that low-income populations disproportionately resided in areas with higher population density and a more compact urban form ([32]; this review did not examine the impact of this relationship on walking and cycling outcomes). On the other hand, it diverges from findings by Cerin et al. [33], who found that the built environment contributed to higher levels of walking for transport among higher educational groups in Australia. One possible explanation of these divergent findings concerns the different type of built environment variables used in the studies: unlike the present study, Cerin et al. included ‘micro’ variables (which are smaller in scale and generally changeable more rapidly and with less cost; see [34] for a description of micro and macro built environment characteristics) like aesthetics, traffic load, physical barriers to walking, and the presence of separate footpaths.

Cycling for transport, walking in leisure time and cycling in leisure time were all significantly more prevalent among respondents with higher educational levels. Adjustment for built environment variables had no or only minimal effect on these educational inequalities. This lack of effect of objective built environment characteristics differs from findings by previous studies, which found objective built environment variables to be a significant contributor to socioeconomic inequalities in walking in leisure time [35], cycling in leisure time [36], and overall physical activity [37, 38]. However, the abovementioned studies again focused mostly on ‘micro’ built environment variables like aesthetics [36], the accessibility of physical activity-facilities (including parks, but also sport centers and youth clubs [37, 38]), and the presence of walking paths [35], whereas the present study used ‘macro’ characteristics such as density, land use pattern and overall accessibility measures. One possible explanation of these differences could be that ‘micro’ characteristics are more likely to be improved in the wealthier, better-organized neighborhoods of higher-educated residents (e.g. it is easier to remove graffiti or design a new bicycle path than to change the density of a neighborhood, and such small changes may take place more often in neighborhoods with high-educated residents). Next to this, the divergent results could be explained by the relatively small educational differences in built environment characteristics (see section “Educational level and built environment”). Although significant effects were found, the small size of these effects suggests that built environment characteristics were quite equally distributed among educational groups. This, together with the compact nature of the Dutch urban environment, might make walking and cycling-promoting resources almost equally accessible to all residents of urban areas.

Previous Dutch studies found that socioeconomic inequalities in recreational walking and cycling could partly be attributed to less favorable built environment characteristics in more disadvantaged neighborhoods [39, 40], which seems in contrast with our results. There are two possible explanations for these differences. Firstly, the divergent findings could be explained by the nature of the neighborhood variables included in the analyses. Where the present study used objective built environment variables, the two studies above used perceptions of either participants [40] or municipal professionals [39]. Also, other neighborhood characteristics than built environment characteristics were applied in those previous Dutch studies (e.g. safety, general attractiveness). Secondly, differences in findings could be explained by differences in the operationalization of socioeconomic position. Where one study [39] used the neighborhood socioeconomic environment and the other [40] used both educational attainment and income, this study used only educational level as an indicator of SEP, and as discussed above, only small educational differences in built environment characteristics were found.

This study showed that the residential patterning of different educational groups can also have positive effects on the accessibility of resources that influence health for groups with lower educational levels. This was the case for walking for transport, the variable that was most strongly correlated to built environment characteristics. Although effects were small due to the small magnitude of differences in built environment characteristics, respondents in lower educational groups had better access to built environment characteristics that positively influenced walking for transport. The health benefits for lower educational groups resulting from their higher levels of walking for transport may partly offset the negative effects of other less healthy behaviors (e.g. the negative effects of less cycling for transport and less recreational walking and cycling; [25]).

### Built environment and walking and cycling

Consistent with previous studies [6, 20], a strong association was found between built environment variables within the walkability domain and walking for transport, with a higher chance of walking for transport among respondents of neighborhoods with high levels of address density, population density, connectivity and accessibility of facilities. Contrary to previous findings [6], the chances of walking in leisure time and cycling in leisure time were lower among respondents living in neighborhoods with higher scores on address density, indicating that an environment that is conducive to walking for transport may have adverse effects on recreational walking and cycling. The fact that this negative association was not found for other variables in the walkability domain (e.g. connectivity) shows that the inclusion of complementary variables can show different effects (i.e. using only a single ‘walkability’ variable would not give this detailed results).

Of all walking and cycling variables, walking for transport was most strongly related to the built environment, which is consistent with previous studies’ findings [20]. For the other walking and cycling variables, relationships were less strong. This was especially the case for cycling for transport, where only one built environment variable was found to be significant: cycling for transport was less prevalent in areas with higher population density. One potential explanation is that in these neighborhoods, more facilities are within walking distance, thereby reducing the need to cycle for transport.

It may be that built environment variables not included in the present study could be more important for cycling for transport, walking in leisure time and cycling in leisure time levels (e.g. aesthetics, presence of walking paths), but it is also possible that these activity types are mainly affected by factors outside the built environment (this could still be neighborhood-level characteristics, such as safety or social features). Another explanation is that these activities may largely take place outside the residential neighborhood, and therefore do not show associations with neighborhood characteristics. Cycling in particular can easily reach beyond the residential neighborhood, enabling residents of lower density areas to reach facilities that are further away from their homes.

### Study strengths

This study has combined survey data from the GLOBE study with objective built environment data from other sources, which has given unique insights into the role of objective built environment factors in explaining inequalities in walking and cycling in the Netherlands. Because this study has focused on the urban region of Eindhoven and its surroundings, the associations found concern an entire urban area, including more suburban and rural surroundings, instead of a single city (as studied in most previous research). This has increased both the variation of built environment characteristics investigated in the study and the possibility of generalizability of this study’s results. Having said that, as compared to the situation in for example the US or Australia, the variation in our urban area might still be small. Another major strength of this study was that it included both walking and cycling, both for transport and in leisure time, whereas other studies have often focused on just one or two outcomes. This broad study design made it possible to compare educational level and built environment influences on different types of physical activity, revealing contrary mechanisms. Also, the incorporation of nine objective built environment variables made it possible to examine the effects of a broad array of different built environment types.

### Study limitations

A number of methodological and analytical problems exist that need to be considered when interpreting this study’s findings. First, the neighborhood area and its 1 km radius used as the spatial analysis units in this analysis may not be similar to the geographic area of residents’ daily spatial movements. People do not take into account neighborhood boundaries in their daily activities, and their activity space may be much larger than just the neighborhood. This is expected to be of importance especially for cycling, where larger distances can be traversed more easily. Second, this study included a selection of built environment variables, while other built environment characteristics might also be important (e.g. aesthetics, presence of walking and cycling trails). Third, a gap of a few years existed between the dates of the various data used. Whereas the GLOBE data was collected in 2011, most built environment variables describe the situation in 2013, and the accessibility of green variables describe the situation in 2008. However, the built environment characteristics used are not expected to have changed much in just a few years. Fourth, the mediation analysis used in the study assumes that there is no misspecification of the causal order, no confounding between the exposure, mediators and outcomes, and no interaction between exposure and mediators [31]. Because the study used a cross-sectional research design, claims about causality cannot be made, but the causal order used in the study is in line with theoretical notions [11, 12]. The analyses were also adjusted for potential confounders (i.e. sex, age, employment status), but there may be other confounders which were not controlled for. The assumption of no interaction was met for walking for transport, cycling for transport and cycling in leisure time. For walking in leisure time an effect of exposure-mediator interaction was found between address density and educational level. Therefore, a stratified analysis of these variables was added in the Additional file 1: Appendix.

A final remark should be made about the possibility of generalization of this study’s results. Like other studies that focused on the contribution of the built environment to socioeconomic inequalities in walking and cycling in the Netherlands [39, 40], this study focused on the city of Eindhoven and its surroundings. The specific spatial patterns that characterize this region should be taken into account when generalizing the results to other urban areas. One example is the spatial patterning of socioeconomic groups. Because the city of Eindhoven does not have a historical city center that tends to attract high socioeconomic groups to the extent that other Dutch cities do, higher-educated residents of Eindhoven might tend to live more in the suburban parts of the city, or even in the smaller urban centers. This pattern might make Eindhoven more comparable to American cities. However, because of other general differences (e.g. in overall density or in cycling behavior) findings should be generalized to other contexts with care. Such differences make comparative research in other cities very relevant.

## Conclusion

Lower educational groups were less likely to cycle for transport, and to walk and cycle in leisure time. Some of the objective built environment characteristics of residential areas examined (i.e. address density, population density, accessibility of facilities and accessibility of public green space) were related to these outcomes. However, only minimal contributions of the built environment to educational inequalities in walking and cycling were found. This may indicate that factors that may explain educational inequalities in these activity types should be searched for outside the residential area, or in other environments than the built environment (e.g. the socio-cultural environments). It could also be that smaller-scale (‘micro’) built environment characteristics of the neighborhood (e.g. aesthetics, presences of walking paths) offer a better explanation.

Despite this, the small effect that was found for walking for transport suggests that the spatial patterning of people by educational level can have a diminishing effect on health inequalities. Lower educational groups tend to live in more walkable neighborhoods, which partly explains their higher tendency to walk for transport. Policy makers that aim to reduce health inequalities should be aware of the positive impact of the residential environment of lower educational groups on walking for transport levels. Recent gentrification processes, in which lower educational groups are forced to relocate to the outskirts of the city, might pose a serious threat to this inequalities-reducing mechanism, and new neighborhoods should be designed to be walkable. Future research should shed more light on the causal mechanisms between socioeconomic position, walking and cycling, and the built environment. A specifically important research type is the comparative analysis of different cities, which might elucidate the role of various urban contexts in the relationships between physical activity and the built environment.

## Additional file

**Additional file 1.** Maps of Eindhoven, and correlations between built environment variables.
