# Supplementary material for: The role of the built environment in explaining educational inequalities in walking and cycling among adults in the Netherlands
Source: Int J Health Geogr. 2017 Mar 31;16:10. doi: 10.1186/s12942-017-0083-y (PMC5374661; doi:10.1186/s12942-017-0083-y)
Supplement: Supplementary file 1 — Additional file 1. Maps of Eindhoven, and correlations between built environment variables. [file 12942_2017_83_MOESM1_ESM.docx]

Appendix – The role of the built environment in explaining educational inequalities in walking and cycling in the Netherlands

Daniël C. van Wijk^a,1^, Joost Oude Groeniger^b,2^, Frank J. van Lenthe^b,3^, Carlijn B.M. Kamphuis^a,*^

^a^ Department of Human Geography and Spatial Planning, Faculty of Geosciences, Utrecht University, Heidelberglaan 2, 3584 CS Utrecht, The Netherlands.

^b^ Department of Public Health, Erasmus University Medical Centre, Erasmus University Rotterdam, Wytemaweg 80, 3015 CN Rotterdam, The Netherlands.

^*^ Corresponding author. c.b.m.kamphuis@uu.nl

^1^ d.c.vanwijk1@students.uu.nl

^2^ j.oudegroeniger@erasmusmc.nl

^3^ f.vanlenthe@erasmusmc.nl

Appendix – The role of the built environment in explaining educational inequalities in walking and cycling in the Netherlands

# Table of Contents

[Table of Contents 2](#_Toc460497666)

[Maps 3](#_Toc460497667)

[Test for correlation between built environment variables 9](#_Toc460497668)

[Statified analysis educational level, address density and walking in leisure time 10](#_Toc460497669)

# Maps

All maps were made using the same categories for the built environment variables as the categories that were used in the statistical analyses. Only neighborhoods inhabited by respondents of the dataset are included, and the municipality map of The Netherlands is used as a background. All maps were created using ArcMap 10.1 and the ‘Rijksdriehoeksmeting’ (RD) coordinate system.


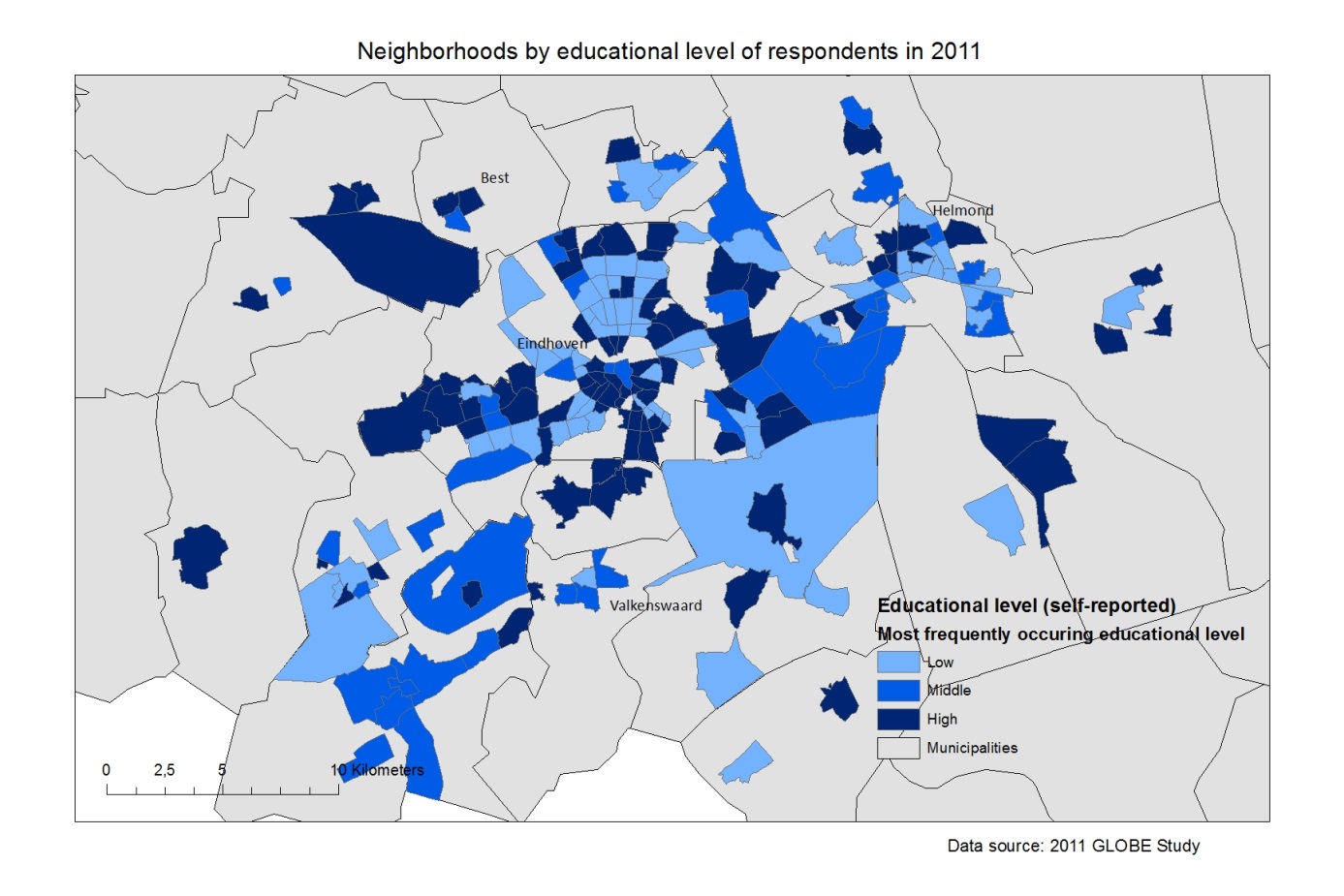


Note: neighborhoods with equal frequencies for two or more of the most frequently occurring educational levels were assigned to the group with the highest educational level (e.g. a neighborhood with 4 low-educated respondents, 4 middle-educated respondents and 2 high-educated respondents was assigned to the ‘middle’ group).


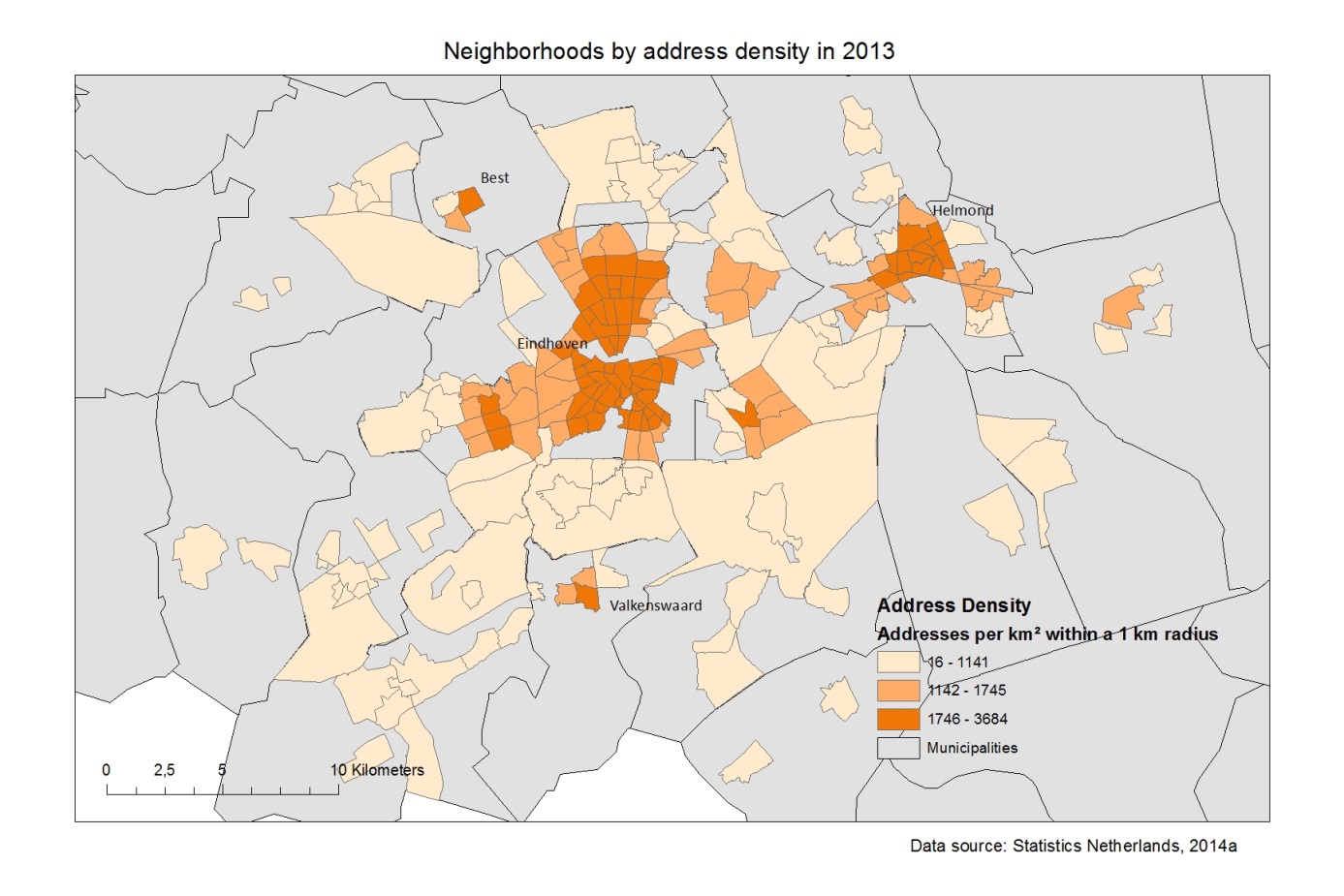


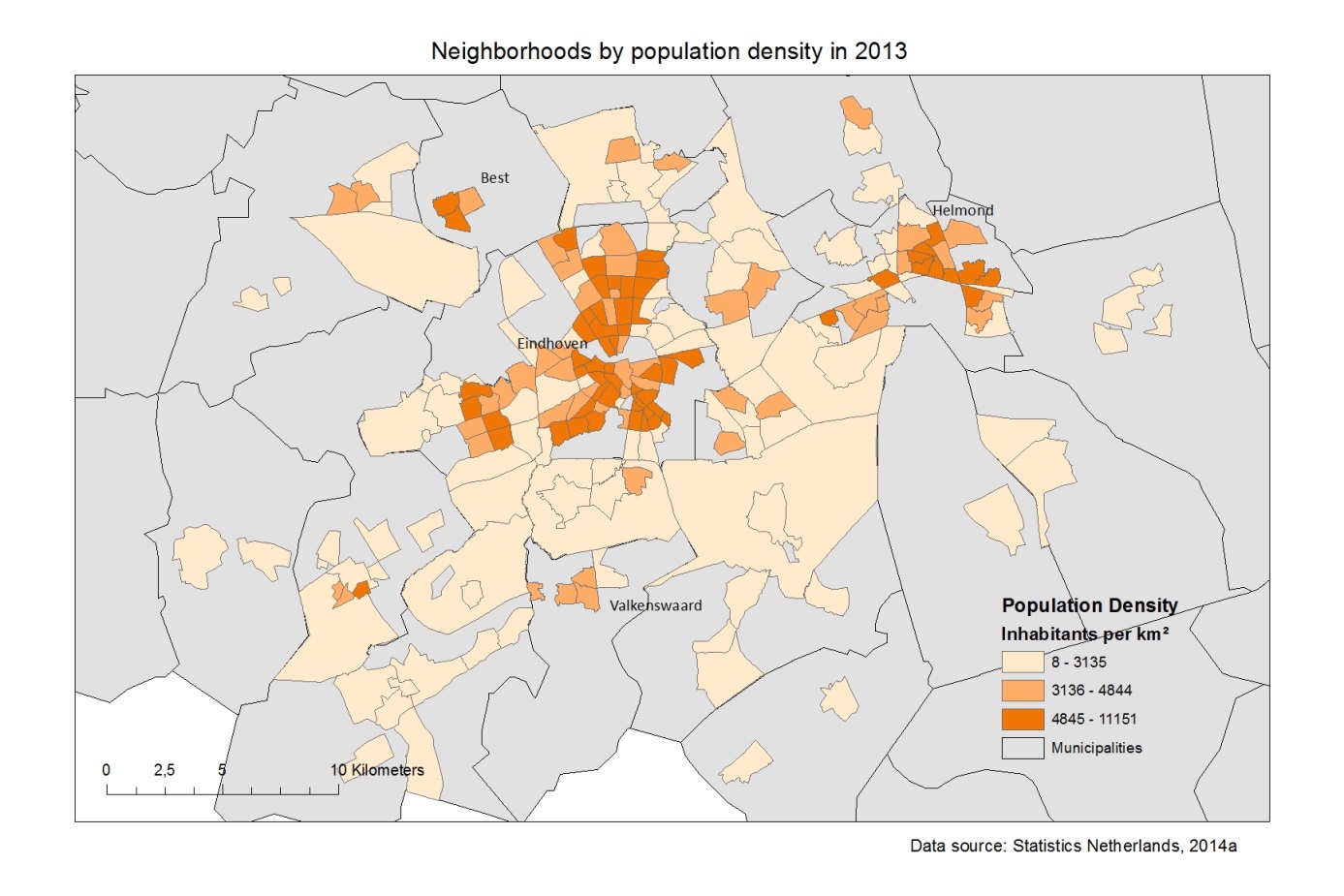


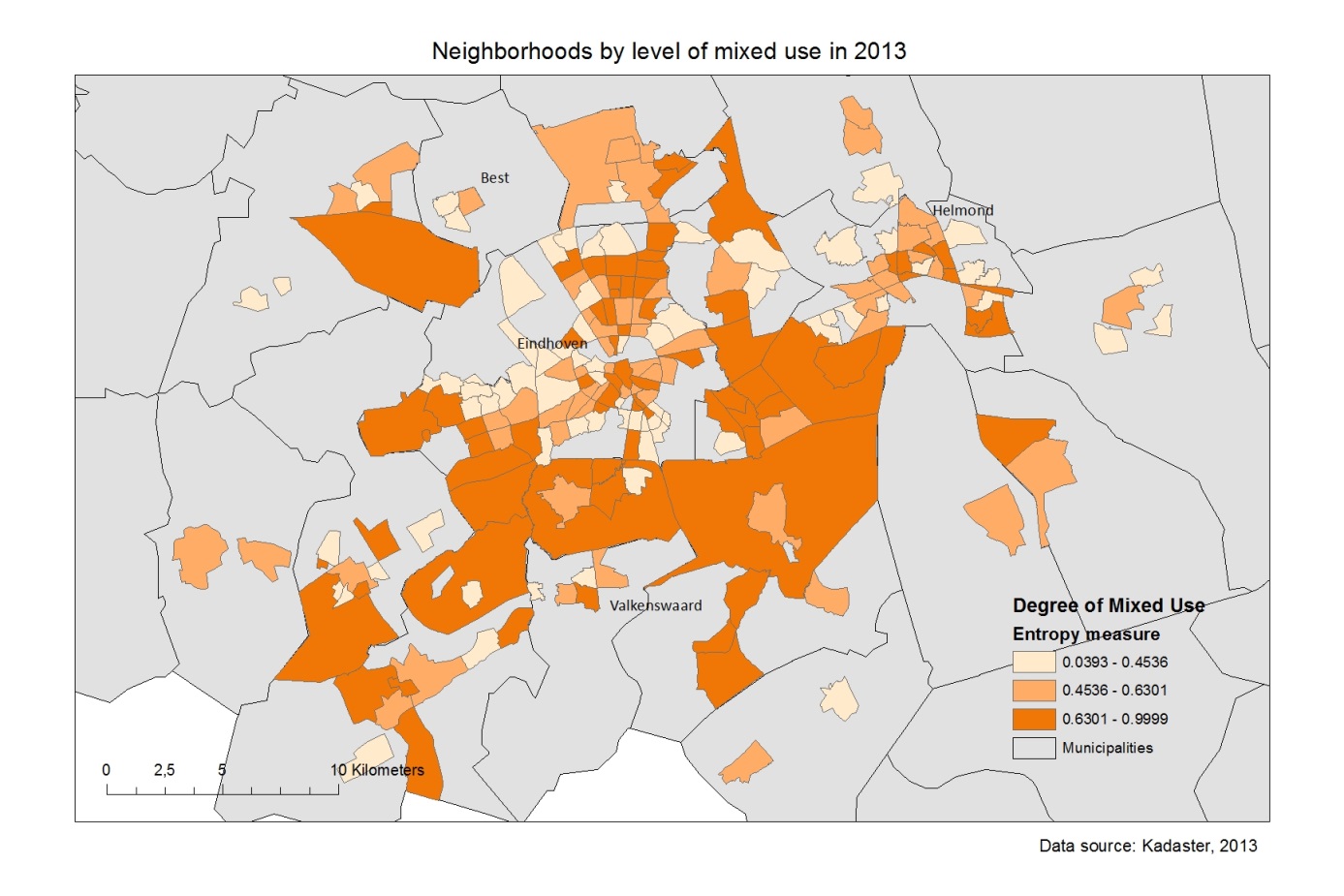


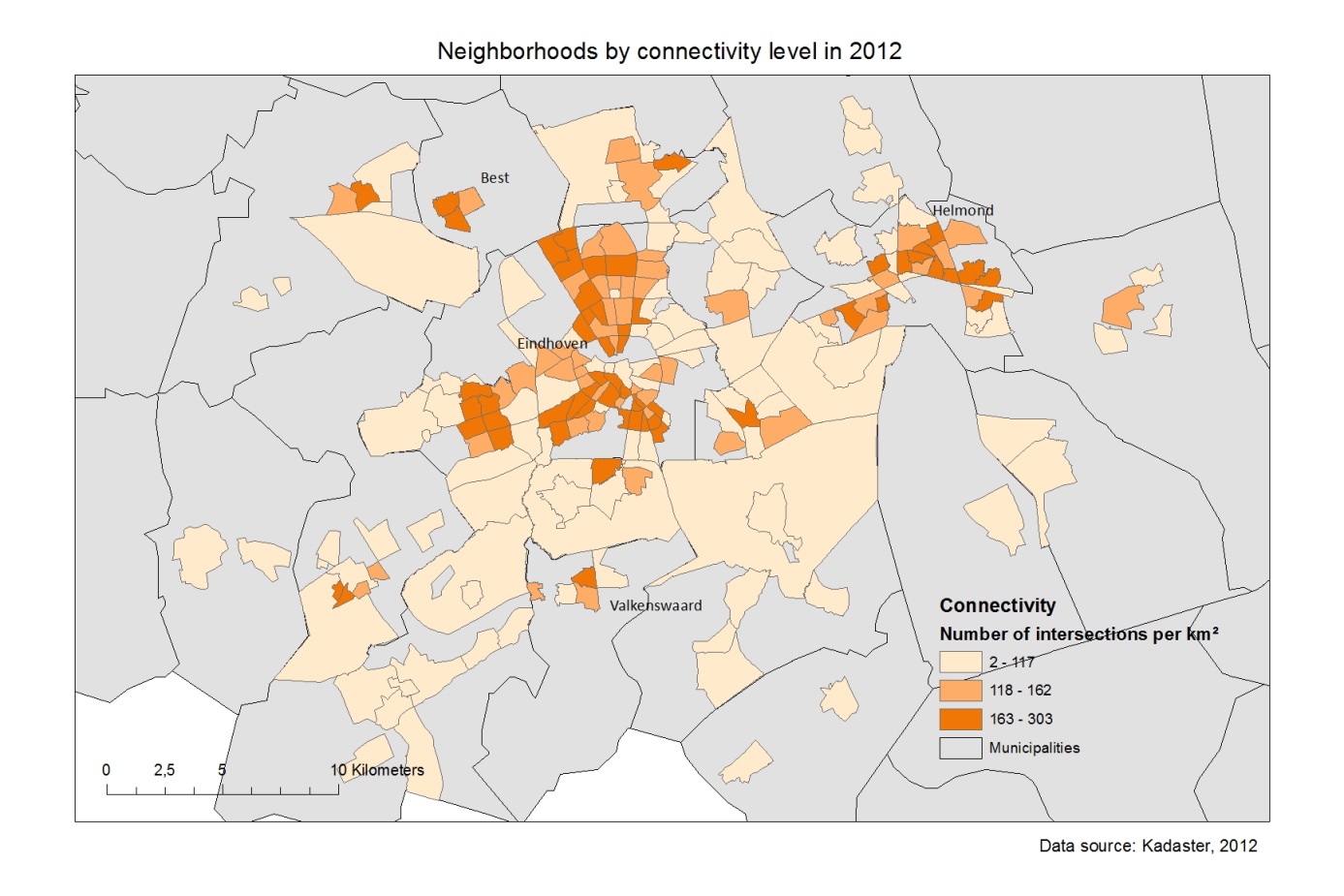


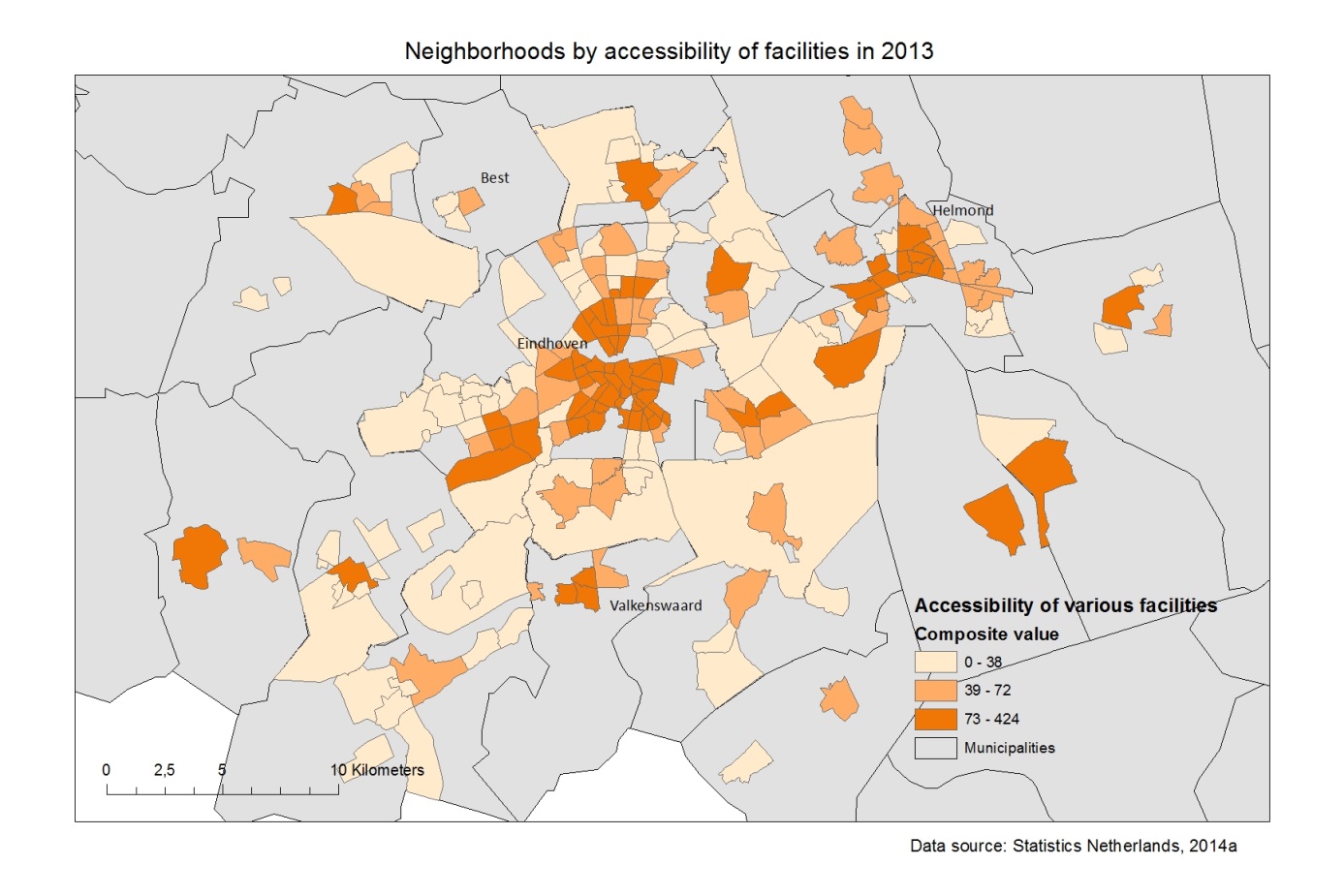


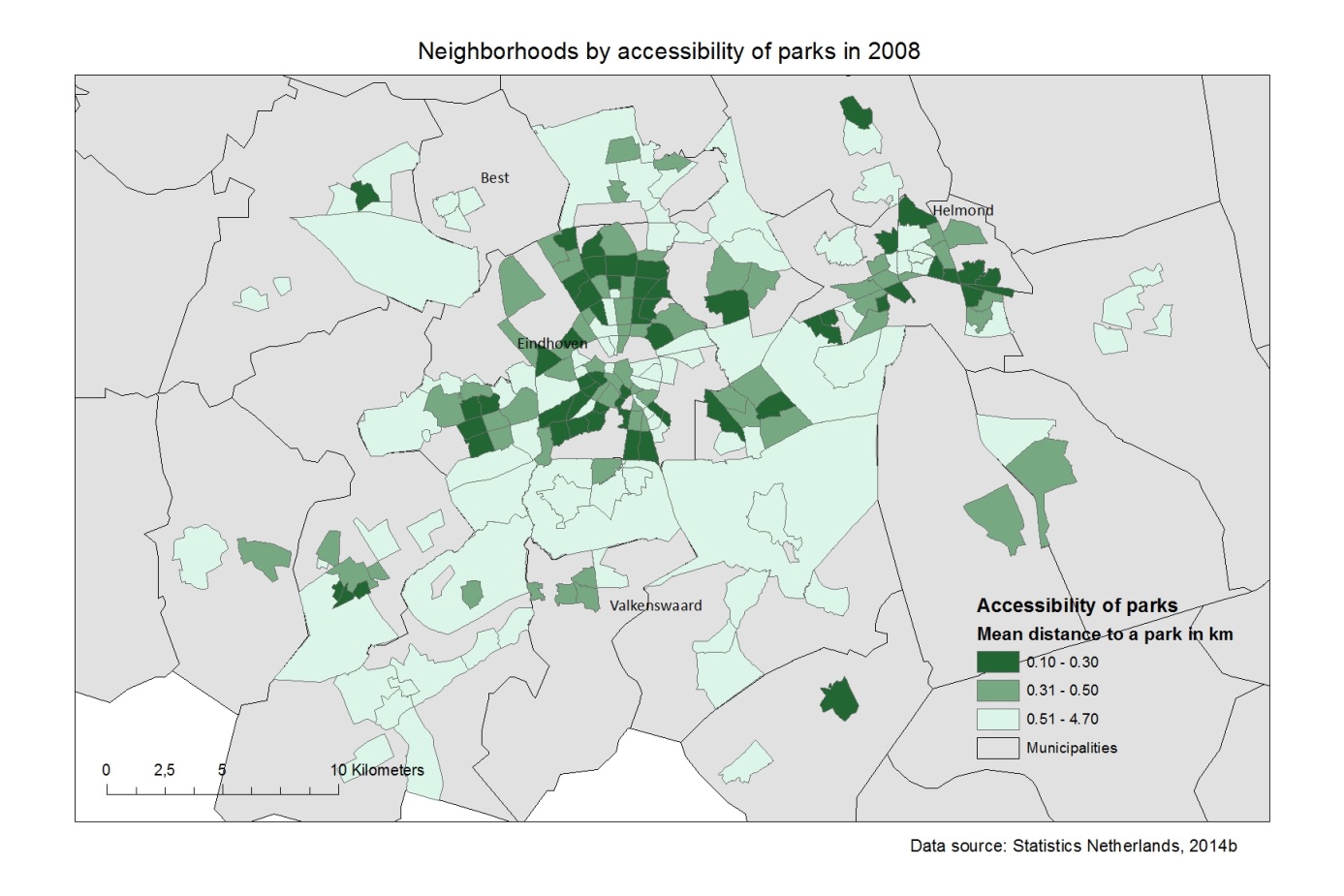


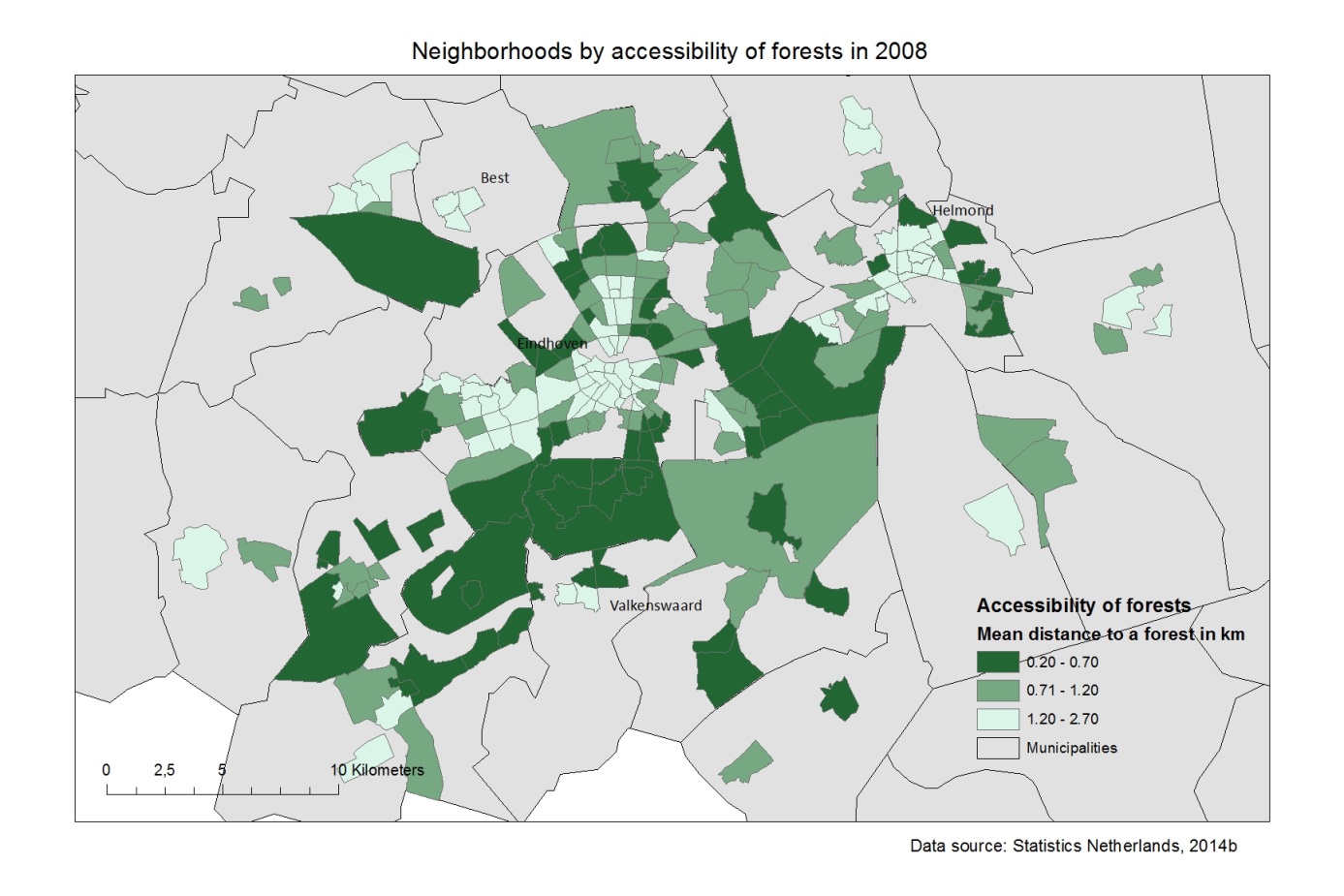


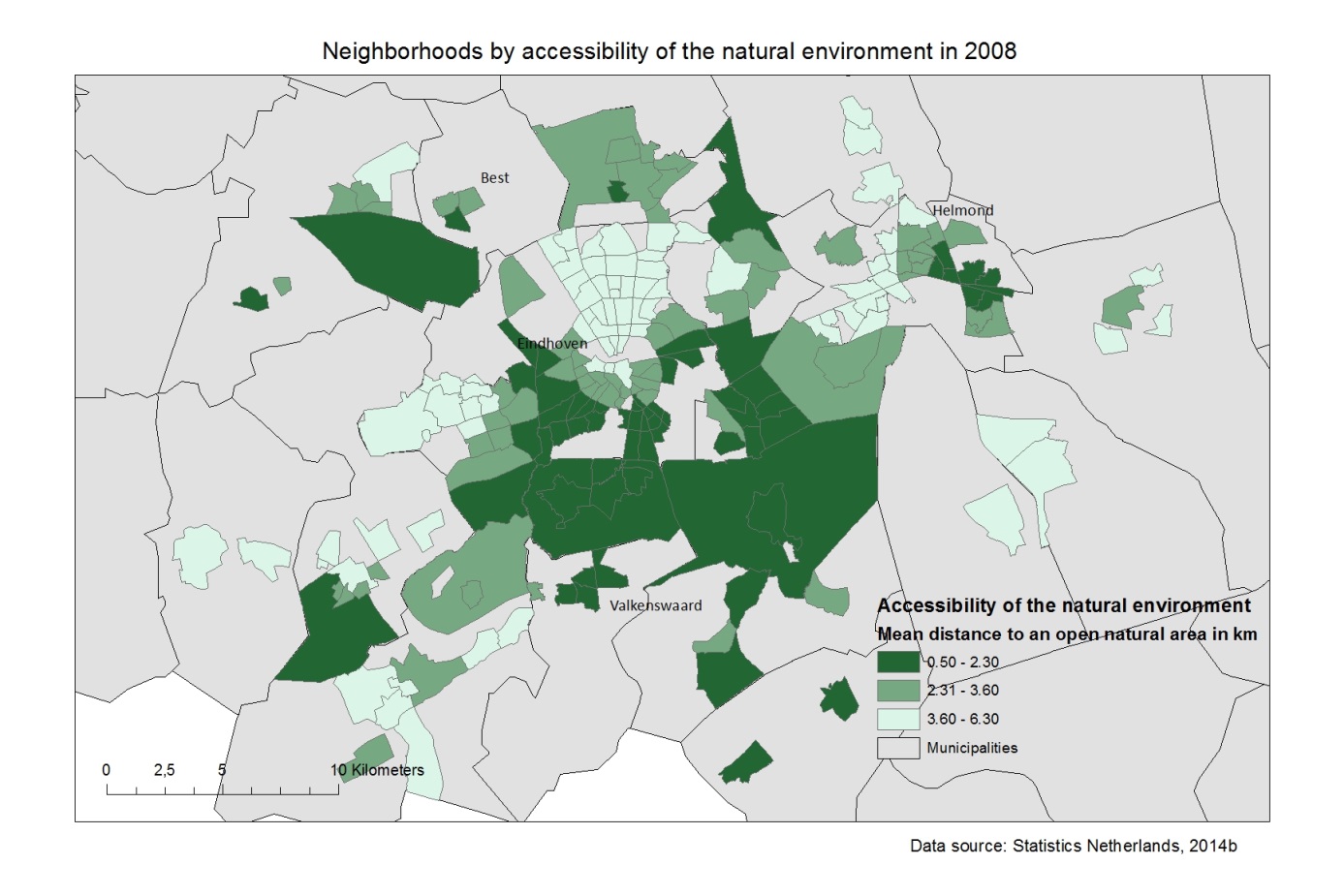


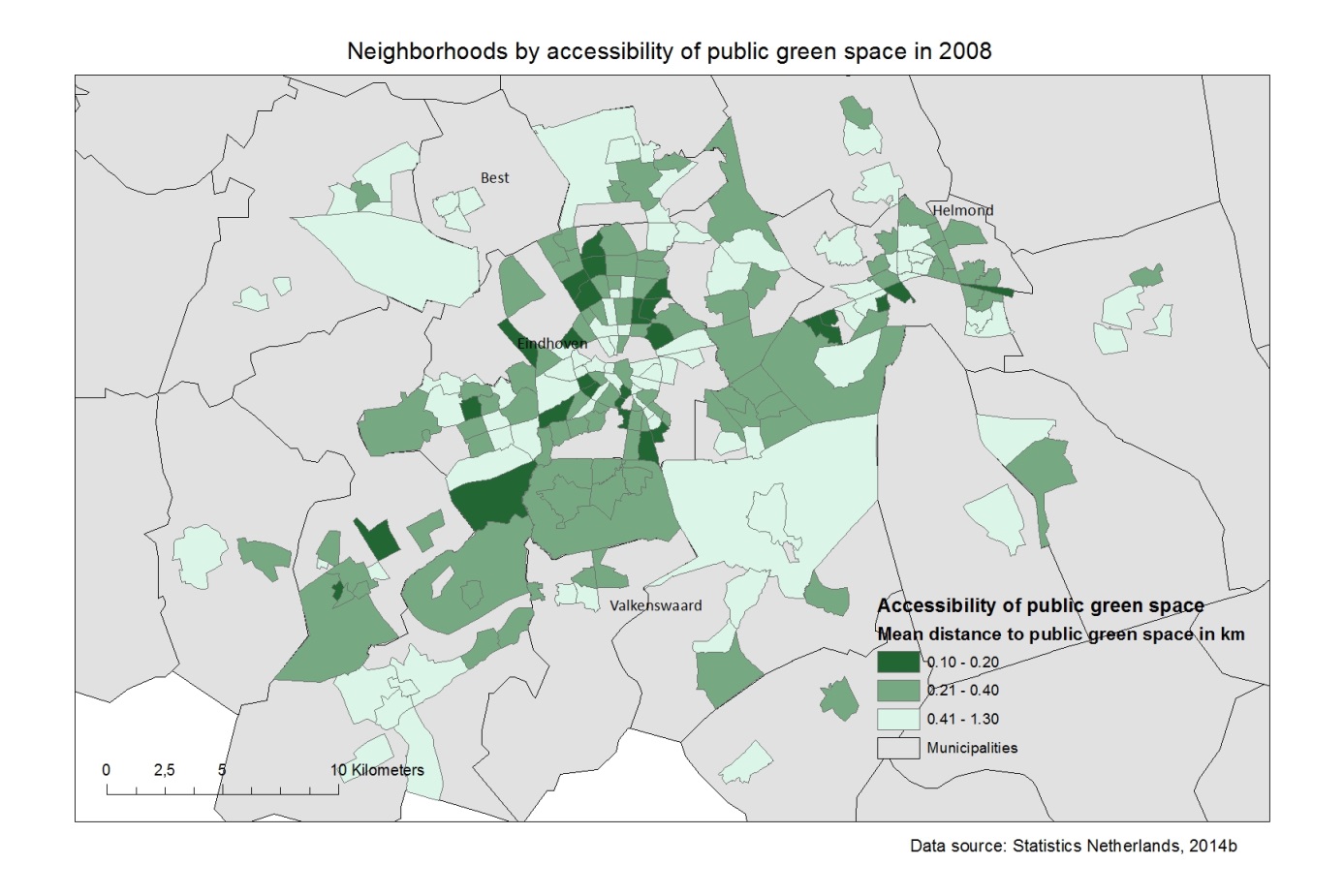


# Test for correlation between built environment variables

Table 1 – Correlation between built environment variables (N=209 neighborhoods)^a^.

|  |  | **Address density** | **Population density** | **Level of mixed use** | **Connectivity** | **Accessibility of facilities** | **Accessibility of parks** | **Accessibility of forests** | **Accessibility of open natural areas** | **Accessibility of public green space** |
| --- | --- | --- | --- | --- | --- | --- | --- | --- | --- | --- |
| **Address density** | **τ_b_** |  | .613 | .059 | .492 | .583 | .278 | -.401 | -.002 | .031 |
|  | **Sig.** |  | .000 | .340 | .000 | .000 | .000 | .000 | .978 | .618 |
| **Population density** | **τ_b_** | .613 |  | -.063 | .626 | .399 | .243 | -.383 | .012 | .040 |
|  | **Sig.** | .000 |  | .312 | .000 | .000 | .000 | .000 | .847 | .514 |
| **Level of mixed use** | **τ_b_** | .059 | -.063 |  | -.148 | .113 | -.112 | -.016 | .020 | -.057 |
|  | **Sig.** | .340 | .312 |  | .017 | .067 | .071 | .796 | .741 | .358 |
| **Connectivity** | **τ_b_** | .492 | .626 | -.148 |  | .278 | .333 | -.273 | .033 | .189 |
|  | **Sig.** | .000 | .000 | .017 |  | .000 | .000 | .000 | .594 | .002 |
| **Accessibility of facilities** | **τ_b_** | .583 | .399 | .113 | .278 |  | .073 | -.366 | .053 | -.123 |
|  | **Sig.** | .000 | .000 | .067 | .000 |  | .240 | .000 | .390 | .047 |
| **Accessibility of parks** | **τ_b_** | .278 | .243 | -.112 | .333 | .073 |  | -.101 | -.049 | .646 |
|  | **Sig.** | .000 | .000 | .071 | .000 | .240 |  | .104 | .431 | .000 |
| **Accessibility of forests** | **τ_b_** | -.401 | -.383 | -.016 | -.273 | -.366 | -.101 |  | .201 | .203 |
|  | **Sig.** | .000 | .000 | .796 | .000 | .000 | .104 |  | .001 | .001 |
| **Accessibility of open natural areas** | **τ_b_** | -.002 | .012 | .020 | .033 | .053 | -.049 | .201 |  | .094 |
|  | **Sig.** | .978 | .847 | .741 | .594 | .390 | .431 | .001 |  | .127 |
| **Accessibility of public green space** | **τ_b_** | .031 | .040 | -.057 | .189 | -.123 | .646 | .203 | .094 |  |
|  | **Sig.** | .618 | .514 | .358 | .002 | .047 | .000 | .001 | .127 |  |

^a^All analyses were conducted using the built environment variables after categorization into tertiles (low, medium, high).

# Statified analysis educational level, address density and walking in leisure time

Table 2 – Associations between educational level and walking in leisure time in different address density categories^a^.

| **Educational level** | **First address density category (N=518)** | | **Second address density category (N=791)** | | **Third address density category (N=1,066)** | | **All categories (N=2,375)** | |
| --- | --- | --- | --- | --- | --- | --- | --- | --- |
|  | **RR (95% CI)** | **Sig.** | **RR (95% CI)** | **Sig.** | **RR (95% CI)** | **Sig.** | **RR (95% CI)** | **Sig.** |
| **Low** | 1.00 | .000 | 1.00 | .069 | 1.00 | .023 | 1.00 | .006 |
| **Middle** | 1.23 (1.10-1.37) |  | 1.04 (0.90-1.21) |  | 1.07 (0.94-1.21) |  | 1.12 (1.04-1.21) |  |
| **High** | 1.04 (0.92-1.18) |  | 1.16 (1.01-1.33) |  | 1.18 (1.04-1.33) |  | 1.12 (1.04-1.21) |  |

^a^ All analyses were adjusted for variations in sex, age and employment status.
